# Within‐Person Seasonal Variability of Aminotransferases and Long‐Term Glycemic Control in Adults With Type 2 Diabetes (JDDM 85)

**DOI:** 10.1111/liv.70743

**Published:** 2026-06-19

**Authors:** Ryota Toki, Masaya Sakamoto, Masahiro Yuki, Miho Iida, Michiko Yamazaki, Seiichi Ichikawa, Ryuzo Horiuchi, Hiroshi Maegawa, Tomonori Okamura, Toru Takebayashi

**Affiliations:** ^1^ Department of Preventive Medicine and Public Health Keio University School of Medicine Shinjuku‐ku Tokyo Japan; ^2^ Department of Diabetes, Metabolism & Endocrinology, School of Medicine International University of Health and Welfare Chiba Japan; ^3^ Department of Diabetes, Metabolism & Endocrinology International University of Health and Welfare Mita Hospital Tokyo Japan; ^4^ Department of Internal Medicine Tsuruoka Kyoritsu Hospital Tsuruoka‐shi Yamagata Japan; ^5^ Department of Clinical Medical Sciences, Graduate School of Medicine International University of Health and Welfare Tokyo Japan; ^6^ Department of Social Medical Sciences, Graduate School of Medicine International University of Health and Welfare Tokyo Japan; ^7^ Patient Engagement Frontier Medical Science KAKEHASHI Inc. Tokyo Japan; ^8^ Department of Cardiology Tsuruoka Kyoritsu Hospital Tsuruoka‐shi Yamagata Japan; ^9^ Department of Pathology Tsuruoka Kyoritsu Hospital Tsuruoka‐shi Yamagata Japan; ^10^ Yasu City Hospital Yasu‐shi Shiga Japan

**Keywords:** aminotransferases, cohort studies, glycated haemoglobin, metabolic dysfunction‐associated steatotic liver disease, seasonal variation, type 2 diabetes mellitus

## Abstract

**Background & Aims:**

Aminotransferases are widely used for metabolic dysfunction‐associated steatotic liver disease (MASLD) evaluation, especially in type 2 diabetes mellitus (T2DM). Whether within‐person seasonal variation affects classification near commonly used thresholds or relates to long‐term metabolic outcomes remains unclear.

**Methods:**

This registry‐based cohort analysed monthly aspartate aminotransferase (AST) and alanine aminotransferase (ALT) measurements from 6039 adults with T2DM in the Japan Diabetes Clinical Data Management registry (2014–2020). Classification discordance across the 30 IU/L threshold was compared between winter‐mean and summer‐mean values. Individual seasonal amplitude was derived from seasonal‐trend decomposition of multiply imputed monthly time series. Final glycated haemoglobin (HbA1c) and non‐achievement of HbA1c < 7% were analysed using multivariable regression.

**Results:**

Both AST and ALT showed significant seasonal variation, with the highest values in late autumn to early winter and the lowest in summer (*p* < 0.001). Approximately one in nine patients with borderline ALT values showed discordant winter‐summer classification. Each 1‐SD increase in AST amplitude was associated with 0.06 percentage points higher final HbA1c (95% CI, 0.04–0.08) and higher odds of not achieving HbA1c < 7% (odds ratio, 1.14; 95% CI, 1.08–1.21; *p* < 0.001). ALT amplitude showed a similar but weaker, less consistent association.

**Conclusions:**

AST and ALT exhibited reproducible seasonal variation peaking in late autumn to early winter, with approximately one in nine patients near the MASLD screening threshold reclassified depending on season. Greater seasonal amplitude, especially AST, was independently associated with poorer glycemic control, supporting season‐aware interpretation in routine clinical practice.

## Key Points

- AST and ALT showed reproducible seasonal variation in adults with type 2 diabetes mellitus, peaking in late autumn to early winter and reaching their lowest values in summer.
- Approximately one in nine patients with borderline ALT values near the 30 IU/L MASLD screening threshold were classified differently depending on the season of measurement.
- Greater individual seasonal aminotransferase amplitude was independently associated with poorer end‐of‐follow‐up glycemic control, more robustly for AST than for ALT.
- Season‐aware interpretation of aminotransferases may improve clinical accuracy, particularly near MASLD screening thresholds.

## Lay Summary

In adults with type 2 diabetes, blood liver enzymes (AST and ALT) tend to be higher in late autumn and winter and lower in summer. Because of this seasonal pattern, about one in nine patients with values near the cutoff used for fatty liver screening would be classified as abnormal or normal depending only on when the test was taken. Patients whose enzyme levels swung more across the seasons also had worse long‐term blood sugar control.

**Abbreviations**
ALT: alanine aminotransferase
AST: aspartate aminotransferase
BMI: body mass index
CI: confidence interval
GLP‐1 RA: glucagon‐like peptide‐1 receptor agonist
HbA1c: glycated haemoglobin
IQR: interquartile range
JDDM: Japan Diabetes Clinical Data Management
LMM: linear mixed model
MASLD: metabolic dysfunction‐associated steatotic liver disease
OR: odds ratio
SD: standard deviation
SGLT2i: sodium‐glucose cotransporter‐2 inhibitor
STL: seasonal‐trend decomposition using locally weighted scatterplot smoothing
T2DM: type 2 diabetes mellitus

## 1 Introduction

Aminotransferases, such as aspartate aminotransferase (AST) and alanine aminotransferase (ALT), are well‐established liver biomarkers, traditionally used as indicators of hepatocellular injury. In recent years, their role has expanded considerably in primary and cardiometabolic care, where they serve as first‐line tests for the clinical assessment of metabolic dysfunction‐associated steatotic liver disease (MASLD). This is particularly relevant in type 2 diabetes mellitus (T2DM), where the high prevalence of MASLD and intensified screening recommendations have placed aminotransferases at the forefront of routine monitoring [1].

In parallel, seasonal variation in biomarkers has attracted increasing interest in cardiometabolic medicine. Several traits relevant to cardiovascular and metabolic health, including body weight, blood pressure and glycemic indices, are known to vary by season, with consistent winter and summer differences described across populations [2, 3, 4]. Moreover, the magnitude of within‐person variability in such traits has itself been linked to adverse outcomes, suggesting that the amplitude of fluctuation can carry clinically meaningful information [5]. By comparison, seasonal patterns in liver enzymes have received limited attention, although Miyake and colleagues previously reported population‐level circannual fluctuations in AST and ALT in a large outpatient sample, with winter peaks and summer nadirs [6]. Whether such variation also occurs at the level of individual patients, and whether it carries clinical relevance, has not been examined.

This question is clinically important for two reasons. First, AST and ALT values near commonly used decision thresholds, such as those used for MASLD screening, may be classified differently depending on when they are measured, but the magnitude of this effect at the individual level is unknown. Second, if individual patients differ in the amplitude of their seasonal aminotransferase fluctuation, this amplitude may itself reflect underlying metabolic instability and be associated with longer‐term outcomes, analogous to variability metrics described for blood pressure and glycemia [7].

To address these questions, we used a nationwide registryof Japanese outpatients with T2DM that provides dense, near‐monthly outpatient laboratory data over multiple years, enabling individual‐level quantification of seasonal aminotransferase amplitude. End‐of‐follow‐up glycated haemoglobin (HbA1c) was selected as the longitudinal outcome because it is measured independently of aminotransferases and reflects the bidirectional hepato‐metabolic axis in T2DM, whereas composite liver indices that incorporate aminotransferases would introduce circular dependence with the exposure. Specifically, we aimed to (1) characterize circannual patterns of AST and ALT at the population level, (2) quantify the extent to which seasonal variation alters clinical classification relative to commonly used aminotransferase thresholds, and (3) examine whether patient‐level seasonal aminotransferase amplitude is associated with end‐of‐follow‐up glycemic control.

## 2 Materials and Methods

### 2.1 Study Population and Data Source

This study was approved by the ethics committee of the Japan Diabetes Clinical Data Management (JDDM) Study Group (ID: JDDM2025‐6) and conducted in accordance with the Declaration of Helsinki. Informed consent for registry participation was obtained from all patients. The requirement for additional consent was waived due to the retrospective design, and an opt‐out option was provided per local regulations.

The JDDM is a nationwide research network of diabetes clinics across Japan that collects clinical data from outpatients with T2DM [8, 9]. This study analysed monthly clinical measurements from the registry covering January 2014 to December 2020. Eligible patients were aged 20–80 years at baseline. Patients with severe hepatic dysfunction (AST or ALT exceeding three times the upper limit of normal, or Child‐Pugh class C cirrhosis) had been excluded prior to data extraction and were not part of the analytic dataset. Patients with ≥ 12 consecutive months of missing aminotransferase data were excluded to ensure robust time‐series data. Baseline data included demographics (age and sex), BMI, smoking and drinking status, glucose‐lowering treatments (insulin, sodium‐glucose cotransporter‐2 inhibitors [SGLT2i] and glucagon‐like peptide‐1 receptor agonists [GLP‐1 RA]), duration of diabetes and HbA1c.

### 2.2 Measurements and Variable Processing

Monthly AST and ALT measurements were obtained during routine clinical visits. If multiple measurements were performed in a single month, the values were averaged for that month. Baseline AST and ALT levels were defined as the values measured in January 2014. For glycemic outcome analysis, the first available HbA1c measurement during the observation period was used as baseline, and the last available HbA1c was used as the outcome. Follow‐up time was calculated as the interval in months between these two measurements. AST and ALT data were log‐transformed to reduce skewness before multiple imputation and seasonality analyses.

### 2.3 Statistical Analysis

#### 2.3.1 Multiple Imputation

Missing monthly AST, ALT and BMI values were addressed using multiple imputation by chained equations [10, 11]. Five complete datasets were generated (*m* = 5), each using 50 iterations of predictive mean matching. Age, sex, smoking status, drinking status and baseline BMI were included as predictors. Patient identifiers were not included in order to preserve the empirical distribution of monthly values across the cohort. Baseline and final HbA1c values were analysed as observed and were not imputed. All subsequent analyses were performed on each of the five imputed datasets, with regression coefficients (point estimates and 95% confidence intervals [CIs]) pooled using Rubin's rules [12] and descriptive counts and percentages averaged across imputations.

#### 2.3.2 Assessment of Population‐Level Seasonality

To assess whether average aminotransferase levels varied across the 12‐month period, two complementary approaches were used. First, a random‐intercepts linear mixed model (LMM) [13] was fitted separately for AST and ALT, with month treated as a fixed (categorical) effect and patient identifier as a random intercept to account for within‐subject correlations. Model inferences were based on the Satterthwaite approximation [14] for degrees of freedom. Estimated marginal means were derived for each month, and pairwise contrasts were examined relative to January. Second, cosinor regression with a 12‐month period [15] was performed to assess whether the data followed a sinusoidal pattern. The amplitude (peak‐to‐trough magnitude) and phase (month of peak enzyme value) were derived from the fitted coefficients.

#### 2.3.3 Quantification of Individual‐Level Seasonal Amplitude

Seasonal‐trend decomposition using locally weighted scatterplot smoothing (STL) [16] was applied to each patient's time series of log‐transformed AST and ALT levels. For each individual, the difference between the maximum and minimum seasonal components represented the individual's amplitude (peak‐to‐trough). This procedure was repeated for each of the five imputed datasets, and the amplitudes were averaged to produce a representative amplitude for each patient. A similar method was used to calculate the BMI amplitude for sensitivity testing.

#### 2.3.4 Analysis of Patient Characteristics by Seasonal Amplitude

To examine whether patients with greater seasonal aminotransferase fluctuations differed in baseline characteristics, we stratified the cohort into quartiles of mean AST and ALT amplitude (averaged across the five imputed datasets) and compared baseline demographic and clinical characteristics across quartiles. Baseline values for this comparison were drawn from the first imputed dataset. Continuous variables were compared using the Kruskal–Wallis test, and categorical variables were compared using Pearson's chi‐square test or Fisher's exact test when expected cell counts were small.

#### 2.3.5 Threshold Crossing and Within‐Person Classification Discordance

To examine the clinical implications of seasonal variation near commonly used decision thresholds, we used a cutoff of 30 IU/L for both AST and ALT, in line with the AASLD 2023 practice guidance, which recommends that elevated ALT or AST above 30 U/L, regardless of sex, may suggest the presence of chronic liver injury [17]. We fitted mixed‐effects logistic regression models with patient as a random intercept, separately using season (summer as reference) and calendar month as predictors. Seasons were defined as winter (December to February), spring (March to May), summer (June to August) and autumn (September to November).

For each patient, the mean of all available winter and summer measurements was computed and classified as abnormal (> 30 IU/L) or normal. Within‐person classification discordance was defined as a difference between the winter‐mean and summer‐mean classifications, regardless of direction, and was assessed in all patients with measurements in both seasons and in a borderline subgroup (annual mean 20–40 IU/L), defined separately for AST and ALT. As ALT thresholds are context‐dependent, we additionally performed sensitivity analyses using sex‐specific ALT thresholds of 30 IU/L for men and 19 IU/L for women, repeating both the seasonal classification and within‐person discordance analyses.

#### 2.3.6 Regression Models for Glycemic Outcomes

We examined whether larger seasonal AST/ALT amplitudes were associated with higher final HbA1c levels using three linear regression models with multiple imputation (*m* = 5):

1. Model 1 (core): Final HbA1c regressed on standardized AST/ALT amplitude (1 SD = 1 unit), baseline AST/ALT (log‐transformed, January 2014), age, sex, baseline HbA1c, BMI and follow‐up duration (months).
2. Model 2 (full): Model 1 plus lifestyle factors (alcohol and smoking status) and diabetes duration.
3. Model 3 (medication usage): Model 2 plus baseline use of insulin, SGLT2i and GLP‐1 RA.

We additionally performed multivariable logistic regression using the same three model structures, with non‐achievement of the guideline‐recommended HbA1c target (< 7% [< 53 mmol/mol]) at end of follow‐up (i.e., final HbA1c ≥ 7%) as a binary outcome. To assess dose–response relationships, we categorized seasonal amplitude into quartiles based on log‐transformed values and analysed using the full model (Model 2). Q1 (lowest variation) served as the reference, and quartile‐specific median amplitudes were back‐transformed to IU/L for clinical interpretation. Tests for linear trend were performed by modelling quartiles as a continuous variable.

#### 2.3.7 Sensitivity and Subgroup Analyses

To evaluate whether AST and ALT amplitudes remained independently linked to final HbA1c after adjusting for weight fluctuations, we added a seasonal BMI amplitude term to the full model. Stratified analyses by baseline BMI (< 25 vs. ≥ 25 kg/m^2^) examined whether associations varied by obesity status. This cutoff was selected based on the recognized threshold for overweight in Asian populations. Effect modification was assessed by including interaction terms (seasonal amplitude × BMI group) in the full cohort model. Given reports that SGLT2i affect both liver function and body weight, additional stratification by SGLT2i use during the final year of follow‐up was conducted, as use was rare at baseline.

Additional sensitivity analyses examined methodological robustness and potential alternative explanations for the observed associations. To evaluate data completeness and potential seasonal imbalance in testing density, we summarized patient‐month observation availability for AST and ALT by calendar month and season, together with the calendar distribution of final HbA1c measurements. To address dependence on imputation, we restricted the analysis to participants with at least 42 observed monthly aminotransferase measurements during the 84‐month observation period and, separately, estimated individual seasonal amplitude by fitting a 12‐month cosinor regression to observed log‐transformed monthly values without imputation among participants with at least 12 observed monthly measurements. To examine whether seasonal amplitude added information beyond average enzyme burden, we replaced baseline AST/ALT with the log‐transformed mean AST/ALT level over follow‐up. To address possible confounding by outcome timing, we additionally adjusted for the season of final HbA1c measurement. To reduce temporal ambiguity, we performed a split‐period analysis in which seasonal amplitude was calculated from 2014–2017 and final HbA1c was assessed during 2018–2020.

All analyses were performed using R version 4.1.2, with statistical significance set at *p* < 0.05. Data analysis was conducted from January 2024 to February 2026.

## 3 Results

### 3.1 Study Population

Of 9949 eligible patients with type 2 diabetes, 3910 were excluded due to ≥ 12 consecutive months of missing aminotransferase data, leaving 6039 patients for the final analysis (Table 1). The average age was 62.9 years (SD 9.9), 61% were male, and the average duration of diabetes was 12.6 years (SD 8.6). Median baseline AST and ALT were both 22 IU/L (IQR: 19–28 and 16–31, respectively). Excluded patients had a slightly higher proportion of males but were otherwise similar in age, BMI and HbA1c (Table S1). Baseline characteristics were comparable before and after multiple imputation (Table S2). Monthly observation availability was balanced across seasons, ranging from 42.9% to 44.0% for AST and from 43.3% to 44.5% for ALT. Final HbA1c measurements were concentrated in late autumn and winter, with 88.7% obtained in November or December (Table S3).

**TABLE 1:** Baseline characteristics of the study population (observed values, *n* = 6039).

Characteristic (Latitude variation of hospitals (degrees North))	Value (26°12′44″–43°11′46″)
Age (years)	62.9 (9.9)
Sex	
Male	3668 (61%)
Female	2371 (39%)
Diabetes duration (years)	12.6 (8.6)
Missing	64
Current alcohol drinking (yes)	1534 (26%)
Missing	76
Current smoking (yes)	855 (14%)
Missing	76
Baseline AST (IU/L)	22 [19, 28]
Missing	3106
Baseline ALT (IU/L)	22 [16, 31]
Missing	3054
Baseline BMI (kg/m^2^)	25.5 (4.2)
Missing	1742
Baseline systolic BP (mmHg)	128.9 (15.2)
Missing	1734
Baseline diastolic BP (mmHg)	75.7 (12.8)
Missing	1734
Baseline HbA1c, % (mmol/mol)	7.2 (1.0) [55 (11)]
Missing	1715
Baseline casual glucose level (mg/dL)	156.1 (56.5)
Missing	2359
Antihypertensive therapy (yes)	3031 (50%)
Insulin therapy (yes)	1240 (21%)
GLP‐1 RA use (yes)	129 (2.1%)
SGLT2i use (yes)	8 (0.1%)
Follow‐up period (months)	81.8 (2.7)
Time from baseline to first HbA1c measurement (months)	0.4 (1.0)
First measured HbA1c, % (mmol/mol)	7.2 (1.1) [55 (12)]
Final measured HbA1c, % (mmol/mol)	7.2 (0.9) [55 (10)]

*Note:* Data are presented as the mean (SD), *n* (%), or median [Q1, Q3]. ‘Missing’ indicates the number of patients lacking data for that variable. Data represent observed (pre‐imputation) values. Imputed baseline characteristics are shown in Table S2.

Abbreviations: ALT, alanine aminotransferase; AST, aspartate aminotransferase; BMI, body mass index; BP, blood pressure; GLP‐1 RA, glucagon‐like peptide‐1 receptor agonist; HbA1c, haemoglobin A1c; SGLT2i, sodium‐glucose cotransporter‐2 inhibitor.

### 3.2 Circannual Seasonality of Aminotransferases

**FIGURE 1:**
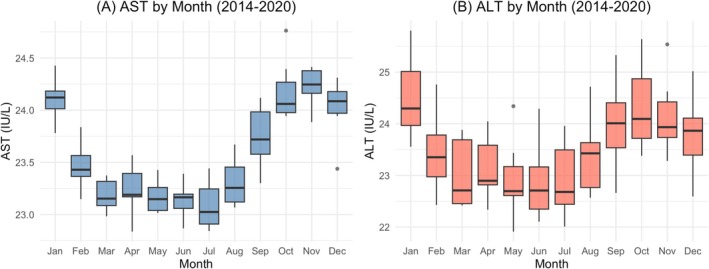
Monthly distribution of AST and ALT levels during 2014–2020. Box plots show the distribution of monthly mean aminotransferase values across the seven‐year observation period for (A) AST and (B) ALT. Each box represents the distribution of seven monthly mean values (one per year from 2014 to 2020). The central line indicates the median, the box represents the interquartile range (IQR), and whiskers extend to the most extreme values within 1.5 × IQR. Both enzymes showed consistent seasonal patterns with peaks in late autumn to early winter and troughs in summer that recurred year after year. Values are presented on the IU/L scale. ALT, alanine aminotransferase; AST, aspartate aminotransferase; IQR, interquartile range.

Monthly mean values of AST and ALT showed distinct seasonal patterns during the 2014–2020 observation period (Figure 1). Both enzymes tended to be highest in late autumn to early winter and lowest in summer, with ALT displaying a more pronounced seasonal variation than AST. These patterns were supported by two complementary analyses. In random‐intercept LMMs with month as a fixed factor, the overall effect of month was highly significant for both AST and ALT (*p* < 0.001; Table 2; Figure S1). The 12‐month cosinor regressions confirmed sinusoidal seasonal fluctuations in both enzymes (*p* < 0.001; Table S4), with estimated peaks in late autumn to early winter and troughs in mid‐summer (Figure S2).

**TABLE 2:** Seasonal variation of AST and ALT Levels: linear mixed‐effects model results.

Month	AST			ALT		
	Beta	95% CI	*p*	Beta	95% CI	*p*
1	*Ref*.	*Ref*.	*Ref*.	*Ref*.	*Ref*.	*Ref*.
2	−0.022	−0.026, −0.018	**< 0.001**	−0.041	−0.047, −0.036	**< 0.001**
3	−0.033	−0.037, −0.028	**< 0.001**	−0.058	−0.063, −0.052	**< 0.001**
4	−0.033	−0.037, −0.029	**< 0.001**	−0.053	−0.059, −0.048	**< 0.001**
5	−0.034	−0.038, −0.030	**< 0.001**	−0.061	−0.067, −0.056	**< 0.001**
6	−0.036	−0.040, −0.032	**< 0.001**	−0.065	−0.071, −0.060	**< 0.001**
7	−0.038	−0.042, −0.034	**< 0.001**	−0.063	−0.069, −0.057	**< 0.001**
8	−0.030	−0.034, −0.026	**< 0.001**	−0.051	−0.056, −0.045	**< 0.001**
9	−0.012	−0.016, −0.008	**< 0.001**	−0.026	−0.032, −0.020	**< 0.001**
10	0.002	−0.002, 0.006	0.118	−0.014	−0.019, −0.008	**0.032**
11	0.008	0.004, 0.012	**< 0.001**	−0.016	−0.022, −0.011	**0.001**
12	0.000	−0.005, 0.004	0.717	−0.029	−0.035, −0.023	**< 0.001**

*Note:* Month 1 (January) is set as the reference month. The beta coefficients represent the estimated difference in AST or ALT levels on a log‐transformed scale relative to those in month 1. Bold values indicate statistical significance (*p* < 0.05).

Abbreviations: ALT, alanine aminotransferase; AST, aspartate aminotransferase; CI, confidence interval.

### 3.3 Patient‐Level Variation in Seasonal Amplitude

Patients were stratified by quartiles based on seasonal amplitude from STL decomposition. Compared to those in the lowest quartile, patients in the highest quartile of AST amplitude were younger (62.3 vs. 63.5 years, *p* = 0.011), more often male (63% vs. 57%, *p* < 0.001), and had higher baseline BMI (26.1 vs. 25.1 kg/m^2^, *p* < 0.001). They also exhibited higher levels of HbA1c, AST and ALT (median AST 25 vs. 20 IU/L; median ALT 26 versus 19 IU/L; both *p* < 0.001), as shown in Table S5. Conversely, ALT amplitude showed fewer demographic differences across quartiles, except for higher baseline AST and ALT levels in the highest quartile (Table S6).

### 3.4 Threshold Crossing and Within‐Person Classification Discordance

**FIGURE 2:**
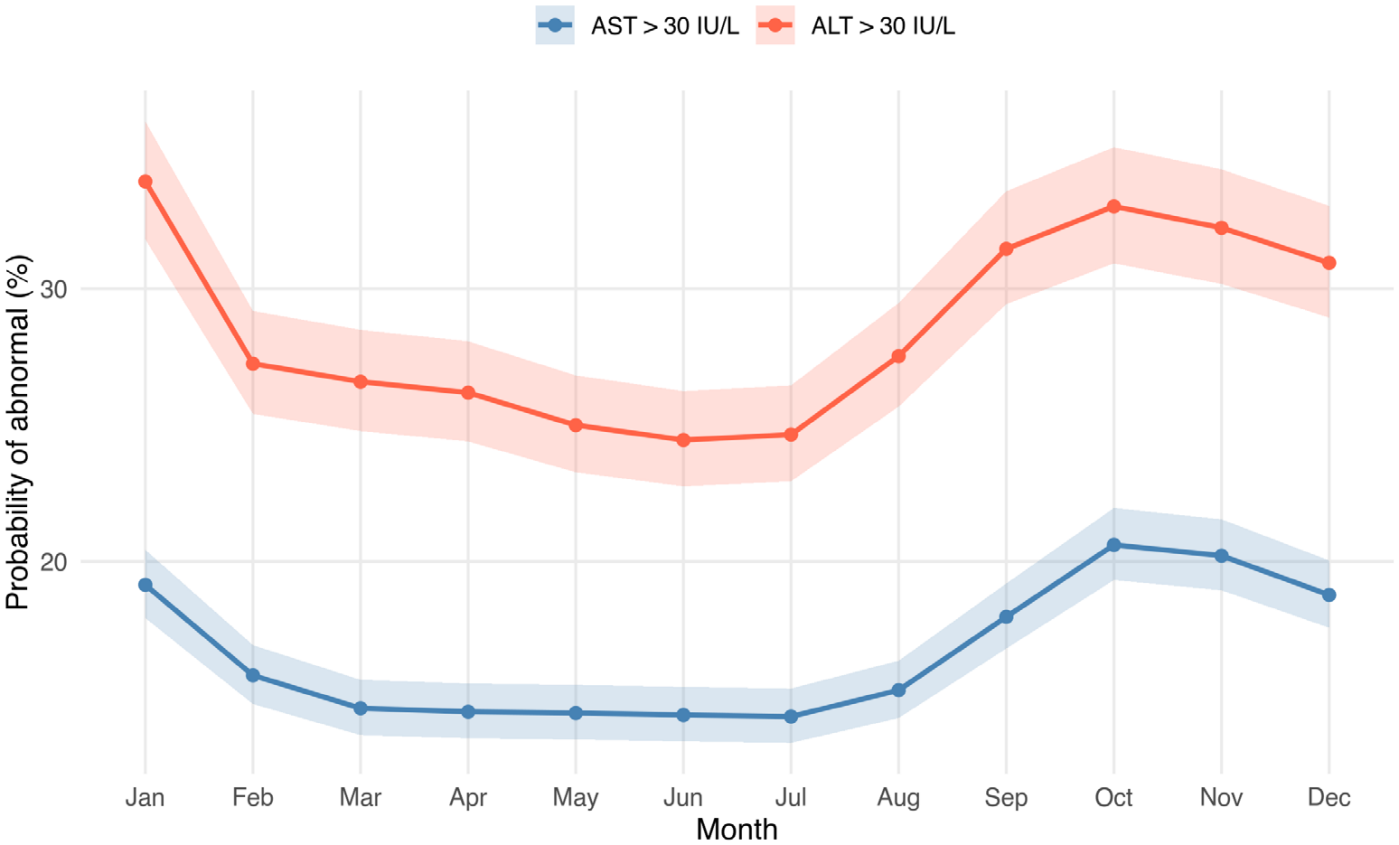
Monthly probability of abnormal aminotransferase values estimated from mixed‐effects logistic regression. Population‐averaged (marginal) probabilities of an abnormal aminotransferase value (> 30 IU/L) by calendar month, derived from mixed‐effects logistic regression models with patient as a random intercept. Marginal probabilities were obtained using bias‐adjustment in the emmeans package to account for the random‐intercept variance, yielding estimates comparable to the observed seasonal rates in Table 3. AST is shown in blue and ALT in red. Solid lines connect monthly point estimates and shaded areas represent 95% confidence intervals. Estimates were obtained from each of the five multiply imputed datasets and averaged. The cutoff of 30 IU/L corresponds to the threshold recommended in the AASLD 2023 practice guidance for the assessment of chronic liver injury. Both enzymes showed a clear seasonal pattern, with the highest probability of abnormal values in autumn to early winter and the lowest in summer. ALT, alanine aminotransferase; AST, aspartate aminotransferase; CI, confidence interval.

To examine whether seasonal variation affected aminotransferase classification near commonly used decision thresholds, we applied a cutoff of 30 IU/L. The probability of an abnormal value varied by month and season for both AST and ALT (Figure 2). In mixed‐effects logistic regression with summer as the reference, the odds of an abnormal AST were higher in autumn (odds ratio [OR] 1.40; 95% confidence interval [CI], 1.35, 1.45; *p* < 0.001) and winter (OR 1.26; 95% CI, 1.21, 1.30; *p* < 0.001), with no difference in spring (*p* = 0.614). Corresponding ORs for ALT were 1.34 (95% CI, 1.27, 1.41) for autumn and 1.26 (95% CI, 1.20, 1.32) for winter, both *p* < 0.001. The abnormal rate ranged from 15.0% in summer to 17.7% in autumn for AST, and from 20.6% to 23.2% for ALT (Table 3). Within‐person classification was discordant between winter‐mean and summer‐mean values in 254 patients (4.2%) for AST and 303 patients (5.0%) for ALT (Table 4). All discordant patients had annual mean values within the borderline range of 20–40 IU/L, indicating that seasonal threshold crossing occurred exclusively in patients whose typical aminotransferase levels were close to the decision cut‐off. Within this borderline subgroup, the discordance rate was 6.7% for AST (254 of 3781) and 11.4% for ALT (303 of 2672). In a sensitivity analysis using sex‐specific ALT thresholds (30 IU/L for men and 19 IU/L for women), the seasonal pattern of abnormal ALT was materially unchanged (Table S7), and the within‐person discordance rate in the borderline subgroup remained similar at 11.7% (Table S8).

**TABLE 3:** Seasonal variation in abnormal aminotransferase rates.

Season	AST > 30 IU/L				ALT > 30 IU/L			
	Abnormal, %	OR	95% CI	*p*	Abnormal, %	OR	95% CI	*p*
Summer	15.0	*Ref*.	*Ref*.	*Ref*.	20.6	*Ref*.	*Ref*.	*Ref*.
Spring	15.0	0.99	0.95, 1.03	0.614	20.8	1.02	0.99, 1.05	0.201
Autumn	17.7	1.40	1.35, 1.45	< 0.001	23.2	1.34	1.27, 1.41	< 0.001
Winter	16.8	1.26	1.21, 1.30	< 0.001	22.6	1.26	1.20, 1.32	< 0.001

*Note:* Winter: December–February; Spring: March–May; Summer: June–August; Autumn: September–November. Summer is set as the reference season. Odds ratios (OR) and 95% confidence intervals (CI) were estimated from mixed‐effects logistic regression with patient as a random intercept, modelling the probability of an aminotransferase value exceeding the cutoff. Estimates were pooled across five multiply imputed datasets using Rubin's rules. Abnormal was defined as aminotransferase > 30 IU/L, based on the AASLD 2023 practice guidance for the assessment of chronic liver injury.

Abbreviations: ALT, alanine aminotransferase; AST, aspartate aminotransferase; CI, confidence interval; OR, odds ratio.

**TABLE 4:** Within‐person discordance in aminotransferase classification between winter and summer.

Classification	AST > 30 IU/L, *n* (%)	ALT > 30 IU/L, *n* (%)
All patients	*n* = 6039	*n* = 6039
Concordant normal	5120 (84.8)	4678 (77.5)
Concordant abnormal	665 (11.0)	1057 (17.5)
Discordant classification	254 (4.2)	303 (5.0)
Borderline subgroup (annual mean 20–40 IU/L)	*n* = 3781	*n* = 2672
Discordant classification	254 (6.7)	303 (11.4)

*Note:* For each patient, the mean of all available winter (December–February) and summer (June–August) aminotransferase measurements across the entire observation period was computed, and each seasonal mean was classified as abnormal (> 30 IU/L) or normal. Only patients with measurements in both seasons were included. Counts and percentages were averaged across five multiply imputed datasets. Concordant normal: both seasonal means ≤ 30 IU/L. Concordant abnormal: both seasonal means > 30 IU/L. Discordant: winter‐mean and summer‐mean classifications differed, regardless of direction. The borderline subgroup included patients whose annual mean aminotransferase fell between 20 and 40 IU/L, representing values closest to the decision threshold. Numbers differ between AST and ALT because the subgroup was defined using the annual mean of each enzyme separately. Abnormal was defined as aminotransferase > 30 IU/L, based on the AASLD 2023 practice guidance for the assessment of chronic liver injury.

Abbreviations: ALT, alanine aminotransferase; AST, aspartate aminotransferase.

### 3.5 Association of Amplitude With Final HbA1c

Multivariable linear regression analyses showed that greater seasonal amplitude was significantly associated with higher final HbA1c for both enzymes (Table 5). In the core model, which adjusted for baseline enzyme level, age, sex, first measured HbA1c, BMI and follow‐up duration, each 1‐SD increase in AST amplitude was associated with a 0.06% (0.66 mmol/mol) higher final HbA1c (*β* = 0.06; 95% CI, 0.04, 0.08; *p* < 0.001). This association persisted in the full model, which additionally adjusted for smoking status, drinking status and diabetes duration. It also persisted in the medication model, which additionally included baseline use of insulin, SGLT2i and GLP‐1 RAs. ALT amplitude showed a smaller yet significant association (*β* = 0.02; 95% CI, 0.00, 0.04; *p* = 0.032 in the full model), which also remained stable across adjustment models.

**TABLE 5:** Multivariable regression models examining the association of seasonal enzyme amplitude with final HbA1c.

Group	Characteristic	Model 1 (Core)			Model 2 (Full)			Model 3 (Med Usage)		
		Beta	95% CI^a^	*p*	Beta	95% CI^a^	*p*	Beta	95% CI^a^	*p*
AST models	**AST amplitude (SD)**	**0.06**	**0.04, 0.08**	**< 0.001**	**0.06**	**0.04, 0.08**	**< 0.001**	**0.06**	**0.04, 0.08**	**< 0.001**
	Baseline AST (2014–01)	−0.11	−0.19, −0.02	0.022	−0.09	−0.18, 0.00	0.041	−0.08	−0.17, 0.00	0.062
	Sex (male)	0.05	0.01, 0.10	0.014	0.07	0.02, 0.11	0.004	0.06	0.02, 0.11	0.004
	Age	0.00	−0.01, 0.00	< 0.001	−0.01	−0.01, 0.00	< 0.001	−0.01	−0.01, 0.00	< 0.001
	First measured HbA1c	0.35	0.33, 0.37	< 0.001	0.34	0.32, 0.36	< 0.001	0.32	0.30, 0.34	< 0.001
	Baseline BMI	0.01	0.01, 0.02	< 0.001	0.01	0.01, 0.02	< 0.001	0.01	0.01, 0.02	< 0.001
	Follow‐up duration (months)	0.00	−0.01, 0.00	0.343	0.00	−0.01, 0.00	0.338	0.00	−0.01, 0.00	0.405
	Current alcohol drinking (yes)				0.01	−0.03, 0.06	0.481	0.02	−0.02, 0.06	0.291
	Current smoking (yes)				−0.02	−0.05, 0.02	0.328	−0.01	−0.04, 0.02	0.497
	Diabetes duration				0.01	0.01, 0.01	< 0.001	0.01	0.00, 0.01	< 0.001
	Baseline insulin therapy (yes)							0.21	0.16, 0.27	< 0.001
	Baseline GLP‐1 RA use (Yes)							0.08	−0.07, 0.23	0.276
	Baseline SGLT2i use (yes)							−0.76	−1.37, −0.15	0.015
ALT models	**ALT amplitude (SD)**	**0.02**	**0.00, 0.05**	**0.026**	**0.02**	**0.00, 0.04**	**0.032**	**0.02**	**0.00, 0.04**	**0.046**
	Baseline ALT (2014–01)	−0.02	−0.08, 0.04	0.599	0.01	−0.05, 0.07	0.844	0.02	−0.04, 0.08	0.479
	Sex (male)	0.05	0.01, 0.10	0.022	0.06	0.02, 0.11	0.005	0.07	0.02, 0.11	0.004
	Age	0.00	−0.01, 0.00	< 0.001	−0.01	−0.01, 0.00	< 0.001	−0.01	−0.01, 0.00	< 0.001
	First measured HbA1c	0.35	0.33, 0.37	< 0.001	0.34	0.32, 0.36	< 0.001	0.32	0.30, 0.34	< 0.001
	Baseline BMI	0.01	0.01, 0.02	< 0.001	0.01	0.01, 0.02	< 0.001	0.01	0.01, 0.02	< 0.001
	Follow‐up duration (months)	0.00	−0.01, 0.00	0.361	0.00	−0.01, 0.00	0.352	0.00	−0.01, 0.00	0.417
	Current alcohol drinking (yes)				0.01	−0.03, 0.05	0.507	0.02	−0.02, 0.06	0.303
	Current smoking (yes)				−0.02	−0.05, 0.02	0.319	−0.01	−0.04, 0.02	0.491
	Diabetes duration				0.01	0.01, 0.01	< 0.001	0.01	0.00, 0.01	< 0.001
	Baseline insulin therapy (yes)							0.22	0.16, 0.27	< 0.001
	Baseline GLP‐1 RA use (yes)							0.08	−0.07, 0.23	0.277
	Baseline SGLT2i use (yes)							−0.76	−1.37, −0.15	0.015

*Note:* Three models are displayed for each enzyme: (1) a core model adjusting for baseline enzyme level and key covariates, (2) a more comprehensive model (full) adding additional lifestyle factors, and (3) a medication‐focused model (med usage). The primary independent variables of interest (AST amplitude and ALT amplitude) are highlighted in bold to distinguish them from the covariates included for adjustment.

Abbreviations: ALT, alanine aminotransferase; AST, aspartate aminotransferase; BMI, body mass index; CI, confidence interval; GLP‐1 RA, glucagon‐like peptide‐1 receptor agonist; HbA1c, haemoglobin A1c; SGLT2i, sodium‐glucose cotransporter‐2 inhibitor.

^a^ Beta coefficients (95% CI) represent the change in final HbA1c (%) per 1‐SD increase in enzyme amplitude or a 1‐unit change in the covariate.

Logistic regression analyses confirmed these findings (Table 6). Each 1‐SD increase in AST amplitude was associated with 14% higher odds of not achieving the HbA1c < 7% target (OR, 1.14; 95% CI, 1.08, 1.21; *p* < 0.001), with consistent estimates across all three adjustment models. ALT amplitude showed a modest association (OR, 1.06; 95% CI, 1.00, 1.12; *p* = 0.036 in the full model). Quartile analysis further demonstrated a dose–response relationship for AST amplitude (Table S9). Compared with patients in Q1 (median seasonal variation: 2.9 IU/L), those in Q4 (median: 7.6 IU/L) had 0.15% higher final HbA1c (95% CI, 0.08, 0.21; *p* < 0.001; *p* for trend < 0.001). In contrast, the association for ALT amplitude did not reach statistical significance across quartiles (Q4 vs. Q1: *β* = 0.05; 95% CI, −0.01, 0.11; *p* = 0.080; *p* for trend = 0.081).

**TABLE 6:** Logistic regression models examining the association of seasonal enzyme amplitude with non‐achievement of HbA1c < 7% target.

Group	Characteristic	Model 1 (Core)			Model 2 (Full)			Model 3 (Med Usage)		
		OR	95% CI^a^	*p*	OR	95% CI^a^	*p*	OR	95% CI^a^	*p*
AST Models	**AST amplitude (SD)**	**1.13**	**1.07, 1.20**	**< 0.001**	**1.14**	**1.08, 1.21**	**< 0.001**	**1.14**	**1.07, 1.20**	**< 0.001**
	Baseline AST (2014–01)	0.74	0.60, 0.89	0.002	0.75	0.61, 0.91	0.004	0.76	0.63, 0.93	0.007
	Sex (Male)	1.09	0.97, 1.21	0.147	1.12	1.00, 1.26	0.054	1.12	1.00, 1.26	0.059
	Age	1.00	0.99, 1.00	0.347	0.99	0.99, 1.00	0.011	0.99	0.99, 1.00	0.021
	First measured HbA1c	2.21	2.06, 2.38	< 0.001	2.14	1.99, 2.30	< 0.001	2.07	1.92, 2.22	< 0.001
	Baseline BMI	1.00	0.99, 1.01	0.913	1.00	0.99, 1.02	0.874	1.00	0.99, 1.01	0.944
	Follow‐up duration (months)	1.01	0.98, 1.03	0.639	1.00	0.98, 1.03	0.685	1.01	0.98, 1.03	0.586
	Current alcohol drinking (yes)				1.06	0.96, 1.18	0.252	1.08	0.97, 1.20	0.152
	Current smoking (yes)				0.97	0.89, 1.06	0.494	0.98	0.90, 1.07	0.666
	Diabetes duration				1.02	1.01, 1.02	< 0.001	1.01	1.01, 1.02	< 0.001
	Baseline insulin therapy (yes)							1.60	1.37, 1.85	< 0.001
	Baseline GLP‐1 RA use (yes)							1.09	0.74, 1.62	0.656
	Baseline SGLT2i use (yes)							0.22	0.04, 1.12	0.068
ALT Models	**ALT amplitude (SD)**	**1.06**	**1.00, 1.12**	**0.043**	**1.06**	**1.00, 1.12**	**0.036**	**1.06**	**1.00, 1.12**	**0.051**
	Baseline ALT (2014–01)	0.89	0.78, 1.03	0.113	0.93	0.81, 1.07	0.312	0.96	0.83, 1.10	0.562
	Sex (Male)	1.07	0.96, 1.20	0.219	1.11	0.99, 1.25	0.079	1.11	0.99, 1.25	0.074
	Age	1.00	0.99, 1.00	0.122	0.99	0.98, 1.00	0.003	0.99	0.99, 1.00	0.008
	First measured HbA1c	2.22	2.06, 2.39	< 0.001	2.14	1.99, 2.31	< 0.001	2.06	1.92, 2.22	< 0.001
	Baseline BMI	1.00	0.99, 1.02	0.900	1.00	0.99, 1.02	0.788	1.00	0.99, 1.02	0.929
	Follow‐up duration (months)	1.01	0.98, 1.03	0.604	1.01	0.98, 1.03	0.649	1.01	0.98, 1.03	0.557
	Current alcohol drinking (yes)				1.06	0.95, 1.17	0.277	1.08	0.97, 1.19	0.165
	Current smoking (yes)				0.97	0.89, 1.06	0.495	0.98	0.90, 1.07	0.668
	Diabetes duration				1.02	1.01, 1.02	< 0.001	1.01	1.01, 1.02	< 0.001
	Baseline insulin therapy (yes)							1.61	1.38, 1.87	< 0.001
	Baseline GLP‐1 RA use (yes)							1.09	0.74, 1.62	0.663
	Baseline SGLT2i use (yes)							0.23	0.05, 1.14	0.071

Abbreviations: ALT, alanine aminotransferase; AST, aspartate aminotransferase; BMI, body mass index; CI, confidence interval; GLP‐1 RA, glucagon‐like peptide‐1 receptor agonist; HbA1c, haemoglobin A1c; OR, odds ratio; SGLT2i, sodium‐glucose cotransporter‐2 inhibitor.

^a^ Odds ratios (95% CI) represent the relative odds of final HbA1c ≥ 7% per 1‐SD increase in enzyme amplitude or a 1‐unit change in the covariate. Three models are displayed for each enzyme: (1) a core model adjusting for baseline enzyme level and key covariates, (2) a more comprehensive model (full) adding additional lifestyle factors, and (3) a medication‐focused model (med usage). The primary independent variables of interest (AST amplitude and ALT amplitude) are highlighted in bold to distinguish them from the covariates included for adjustment.

### 3.6 Sensitivity and Subgroup Analyses

The association between aminotransferase amplitude and final HbA1c was independent of seasonal weight fluctuations. After adding seasonal BMI amplitude to the full model, AST amplitude remained associated with final HbA1c (*β* = 0.06%; 95% CI, 0.04, 0.08; *p* < 0.001), and ALT amplitude also remained associated (*β* = 0.02%; 95% CI, 0.00, 0.04; *p* = 0.040), indicating that the observed associations were not driven by parallel seasonal changes in body weight (Table S10).

Effect modification by baseline BMI was observed for both enzymes (*p* for interaction = 0.005 for AST and 0.009 for ALT; Table S11). In the higher BMI group (≥ 25 kg/m^2^, *n* = 3027), both AST amplitude (*β* = 0.10%; 95% CI, 0.06, 0.13; *p* < 0.001) and ALT amplitude (*β* = 0.05%; 95% CI, 0.02, 0.09; *p* = 0.002) were associated with final HbA1c, while in the lower BMI group (< 25 kg/m^2^, *n* = 3012) the association was modest for AST (*β* = 0.03%; 95% CI, 0.00, 0.06; *p* = 0.038) and not detected for ALT (*β* = −0.01%; *p* = 0.626). Stratification by SGLT2i use during the final year of follow‐up showed that AST amplitude remained associated with final HbA1c in both users (*β* = 0.04; 95% CI, 0.00, 0.08; *p* = 0.029) and non‐users (*β* = 0.07; 95% CI, 0.04, 0.10; *p* < 0.001), with similar but non‐significant point estimates for ALT amplitude (*β* = 0.02 in both groups; Table S12).

To further evaluate the robustness of the amplitude–HbA1c associations, we performed several additional sensitivity analyses (Table S13). The AST amplitude association with final HbA1c remained statistically significant across all sensitivity models. This included analyses restricted to participants with at least 42 monthly aminotransferase observations (*β* = 0.080; 95% CI, 0.041, 0.119; *p* < 0.001), observed‐data cosinor analyses without imputation (*β* = 0.047; 95% CI, 0.025, 0.069; *p* < 0.001), replacement of baseline AST with the mean enzyme level over follow‐up (*β* = 0.050; 95% CI, 0.027, 0.073; *p* < 0.001), additional adjustment for the season of final HbA1c measurement (*β* = 0.06; 95% CI, 0.04, 0.08; *p* < 0.001), and a split‐period analysis using amplitude during 2014–2017 and final HbA1c during 2018–2020 (*β* = 0.043; 95% CI, 0.019, 0.067; *p* < 0.001). The corresponding ALT association persisted in the observed‐data cosinor analysis (*β* = 0.026; 95% CI, 0.004, 0.048; *p* = 0.019) but was attenuated after adjustment for mean enzyme level (*β* = 0.010; *p* = 0.344) and in the split‐period analysis (*β* = 0.004; *p* = 0.722).

## 4 Discussion

In this large registry‐based cohort of Japanese outpatients with T2DM, AST and ALT exhibited reproducible circannual cycles over 7 years, with the highest values in late autumn to early winter and the lowest in summer. Beyond population‐level seasonality, we quantified patient‐level seasonal amplitude and demonstrated substantial inter‐individual heterogeneity. This seasonal variation had direct implications for clinical interpretation. Within‐person classification was discordant between winter‐mean and summer‐mean values in approximately one in nine patients with borderline ALT values. Furthermore, greater seasonal amplitude was independently associated with worse glycemic control at the end of follow‐up. Each 1‐SD increase in AST amplitude was associated with 14% higher odds of failing to achieve the HbA1c < 7% target, and ALT amplitude showed a similar but more modest association. These associations remained significant after adjustment for seasonal BMI amplitude, indicating that aminotransferase seasonality reflects metabolic variability beyond what is captured by seasonal weight fluctuations alone. These findings suggest that aminotransferase seasonality has implications for both clinical classification and longitudinal metabolic outcomes whenever these enzymes are interpreted in routine clinical practice, including primary care settings where MASLD screening is typically initiated.

Population‐level seasonal variation in aminotransferases has been described in earlier studies [6], and HbA1c itself shows seasonal fluctuations in multiple populations, including Japan [3, 18, 19]. However, to our knowledge, individual‐level seasonal amplitude of aminotransferases has not been previously quantified or examined in relation to clinical classification or longitudinal metabolic outcomes. Our results extend this literature in two ways. First, we demonstrated that within‐person seasonal variation directly affects how aminotransferase values are classified relative to clinical decision thresholds. Second, we showed that the magnitude of an individual's seasonal amplitude is associated with subsequent glycemic control. Conceptually, seasonal amplitude can be viewed as a within‐person ‘volatility phenotype’, analogous to variability metrics studied in other cardiometabolic domains [5]. While causality cannot be inferred from this observational design, the observed associations suggest that seasonal aminotransferase dynamics capture risk‐relevant information beyond static, single‐time measurements.

Several mechanisms may contribute to the observed winter‐summer aminotransferase cycles. Cold exposure during winter activates sympathetic and brown adipose tissue responses, which stimulate lipolysis in white adipose tissue and increase the flux of free fatty acids into the liver, thereby raising hepatic lipid load and hepatocellular stress [20]. Concurrently, environmental cues such as ambient temperature and photoperiod modulate circadian and circannual biology, influencing peripheral clocks, low‐grade inflammation and systemic insulin sensitivity [4, 21, 22]. Lower vitamin D availability [23] and reduced physical activity in winter [24, 25] may further contribute to hepato‐metabolic strain. In Japan, as in many countries, late autumn and winter also coincide with cultural events such as year‐end gatherings and New Year celebrations, which may transiently increase caloric and alcohol intake while reducing physical activity. Such seasonally clustered behaviours could plausibly contribute to the observed aminotransferase elevation in addition to the biological mechanisms described above. These pathways act on partially distinct tissues, which may explain the differential patterns observed for AST and ALT. ALT is predominantly liver‐derived, whereas AST is also expressed in skeletal muscle and myocardium in addition to the liver [26]. Accordingly, ALT amplitude may primarily reflect changes in hepatic lipid load and adiposity‐related inflammation, consistent with its more pronounced association in patients with BMI ≥ 25 kg/m^2^. AST amplitude, by contrast, may additionally capture seasonal shifts in muscle activity and systemic insulin sensitivity, integrating broader multisystem metabolic fluctuations [27]. Consistent with this interpretation, the observed associations were independent of seasonal BMI fluctuations, which integrate the cumulative weight effects of both biological and behavioural seasonal influences, supporting the view that aminotransferase amplitude reflects mechanisms beyond simple seasonal weight change. From this perspective, the magnitude of an individual's seasonal aminotransferase amplitude may be interpreted as a marker of how robustly the hepato‐metabolic system buffers recurring environmental and metabolic pressures, with greater amplitude reflecting reduced metabolic resilience. This framework offers a plausible link between seasonal variability and the longer‐term glycemic outcomes observed in our analysis.

From a clinical perspective, our findings are relevant to contemporary clinical practice in which aminotransferases are routinely used for MASLD screening, longitudinal monitoring and treatment response evaluation [28, 29], given MASLD's established association with increased mortality [1, 30] and cardiovascular disease risk in T2DM [31]. We found that the probability of an abnormal aminotransferase value (> 30 IU/L) varied by season, with autumn and winter showing higher odds of abnormal values than summer for both AST and ALT. More importantly, within‐person classification was discordant between winter‐mean and summer‐mean values in 4.2% of patients for AST and 5.0% for ALT, with the discordance rising to 6.7% and 11.4%, respectively, in the clinically most relevant subgroup of patients with annual mean values near the decision threshold. In other words, approximately one in nine patients with borderline ALT values would be classified as abnormal or normal depending solely on the season of measurement. Notably, the AASLD 2023 practice guidance recognizes that aminotransferase levels intermittently elevated above the 30 U/L threshold may suggest chronic liver injury [17]. Our findings extend this clinical observation by showing that a substantial portion of such within‐person variability across the threshold is attributable to predictable seasonal changes. This supports the importance of season‐aware interpretation, or season‐matched comparisons, when evaluating individual aminotransferase values near commonly used decision thresholds in routine clinical practice.

Beyond clinical classification, the magnitude of an individual's seasonal amplitude was independently associated with subsequent glycemic control. Patients with greater seasonal AST or ALT amplitude were more likely to have higher final HbA1c and to fail to achieve the recommended target of < 7%, even after adjustment for baseline enzyme levels, demographic factors, lifestyle and seasonal weight fluctuations. Although the absolute HbA1c effect per 1‐SD increment was modest at approximately 0.06 percentage points for AST, the gradient was more pronounced at the extremes of the amplitude distribution. Patients in the highest AST amplitude quartile had 0.15 percentage points higher final HbA1c compared with those in the lowest quartile, and each 1‐SD increment was associated with 14% higher odds of not achieving HbA1c < 7%. This suggests that aminotransferase amplitude may provide information about metabolic instability beyond what is captured by static, single‐time measurements. Whether this marker can be incorporated into routine clinical interpretation or longitudinal monitoring warrants further investigation.

This study has several strengths. First, our analysis was based on dense monthly longitudinal aminotransferase measurements from over 6000 patients with T2DM followed for 7 years across multiple centres, providing a level of within‐person resolution rarely available in observational liver enzyme research. This was made feasible by Japan's universal health insurance system, which provides comprehensive outpatient coverage and ensures that patients with chronic conditions such as T2DM attend regular outpatient visits with concurrent biochemical monitoring, including aminotransferases [32]. Second, this dataset enabled a novel analytical approach. To our knowledge, this is the first study to quantify individual‐level seasonal aminotransferase amplitude and link it to both clinical classification near MASLD screening thresholds and a longitudinal glycemic outcome, addressing two distinct clinical questions within a single cohort. Third, our findings were supported by methodological robustness, including complementary seasonality methods (LMM, cosinor regression and STL decomposition) that yielded convergent evidence, multiple imputation for missing monthly measurements, and a comprehensive set of sensitivity analyses including adjustment for seasonal BMI fluctuations as well as stratification by baseline BMI and SGLT2i use.

Several limitations should be acknowledged. First, and most importantly, several time‐varying variables were not available in the registry. Information on dietary intake and physical activity was not available, and drinking and smoking status were assessed only at baseline and not updated during follow‐up. This leaves residual confounding by these seasonally varying lifestyle factors as a possibility, particularly during late autumn and winter given the cultural patterns described above. Although adjustment for seasonal BMI amplitude partially captures the integrated weight effects of such behaviours, it cannot fully account for direct influences on aminotransferase levels that occur independently of weight. Vital status and reasons for loss to follow‐up were not directly recorded, introducing potential selection bias. However, because our outcomes were continuous metabolic indicators rather than hard clinical endpoints, the impact on the observed associations is likely limited. Second, the study was conducted exclusively in Japanese outpatients with T2DM, and generalizability to other ethnic and clinical settings warrants confirmation. In addition, the absence of a non‐diabetic comparator group precludes determination of whether the observed seasonal patterns and their association with glycemic control reflect T2DM‐specific pathophysiology or normal biological rhythms. While population‐level seasonal variation in aminotransferases has been reported in non‐diabetic outpatients [6], whether individual seasonal amplitude carries similar clinical implications outside T2DM remains to be examined. Third, our multiple imputation model was intentionally specified without patient identifiers as a clustering variable, in order to preserve the empirical seasonal pattern within each calendar month. This may have modestly attenuated individual seasonal amplitudes; however, such attenuation would bias the observed associations toward the null. Finally, although patients with severe hepatic dysfunction were not included in the source data, the registry does not capture aetiology‐specific liver diagnoses, and patients with viral hepatitis, alcohol‐related liver disease, or hepatic malignancy could not be specifically identified. As an observational study, causal inference requires further investigation.

In conclusion, AST and ALT exhibit robust circannual patterns peaking in late autumn to early winter in adults with T2DM, and seasonal variation has direct implications for clinical interpretation. Approximately one in nine patients with borderline ALT values would be classified as abnormal or normal depending solely on the season of measurement. Greater patient‐level seasonal amplitude was also independently associated with poorer glycemic control, with a more robust association observed for AST than for ALT. These findings support season‐aware interpretation of aminotransferases in routine clinical practice, particularly near MASLD screening thresholds, and identify seasonal amplitude as a potential marker of hepato‐metabolic instability. Future studies examining the temporal stability of individual amplitude and its longitudinal association with metabolic outcomes in independent cohorts would further clarify clinical utility.

## Author Contributions

Conceptualization: Ryota Toki, Masaya Sakamoto. Data curation: Ryota Toki, Masahiro Yuki, Miho Iida, Tomonori Okamura. Formal analysis: Ryota Toki, Masaya Sakamoto, Toru Takebayashi. Investigation: Masahiro Yuki, Michiko Yamazaki, Seiichi Ichikawa, Ryuzo Horiuchi, Hiroshi Maegawa. Methodology: Ryota Toki, Masaya Sakamoto. Project administration: Masaya Sakamoto. Resources: Michiko Yamazaki, Seiichi Ichikawa, Ryuzo Horiuchi, Hiroshi Maegawa. Software: Ryota Toki. Supervision: Masaya Sakamoto, Toru Takebayashi, Tomonori Okamura. Validation: Miho Iida, Tomonori Okamura. Visualization: Ryota Toki. Writing – original draft: Ryota Toki, Masaya Sakamoto. Writing – review and editing: all authors. All authors approved the final version of the manuscript and agree to be accountable for all aspects of the work.

## Funding

The authors have nothing to report.

## Ethics Statement

All procedures were performed in accordance with the ethical standards of the responsible committee on human experimentation and with the Declaration of Helsinki. This study was approved by the Ethics Committee of the Japan Diabetes Clinical Data Management (JDDM) Study Group (approval ID: JDDM2025‐6).

## Consent

Informed consent for registry participation was obtained from all patients. The requirement for additional study‐specific consent was waived due to the retrospective design, and an opt‐out option was provided in accordance with local regulations.

## Conflicts of Interest

The authors declare no conflicts of interest.

## Supporting information


**Figure S1:** Estimated marginal means of monthly AST and ALT levels from random‐intercept linear mixed models.
**Figure S2:** Cosinor fitted curves for AST and ALT (12‐month Cycle).
**Table S1:** Baseline characteristics of the included and excluded patients.
**Table S2:** Pre‐ and post‐imputation baseline patient characteristics.
**Table S3:** Calendar distribution of monthly aminotransferase observations and final HbA1c measurements.
**Table S4:** Seasonal variation of AST and ALT levels: Cosinor Regression Results.
**Table S5:** Comparison of baseline patient characteristics across quartiles of seasonal AST amplitude.
**Table S6:** Comparison of baseline patient characteristics across quartiles of seasonal ALT amplitude.
**Table S7:** Sensitivity analysis: seasonal variation in abnormal ALT rates using sex‐specific thresholds.
**Table S8:** Sensitivity analysis: within‐person ALT classification discordance using sex‐specific thresholds.
**Table S9:** Association of seasonal amplitude quartiles of AST and ALT with final HbA1c.
**Table S10:** Sensitivity analysis incorporating seasonal BMI amplitude.
**Table S11:** Association between seasonal amplitude of liver enzymes and final HbA1c stratified by body mass index.
**Table S12:** Stratified analysis by SGLT2 inhibitor use in the final year.
**Table S13:** Sensitivity analyses for the association between seasonal aminotransferase amplitude and final HbA1c.

## Data Availability

The deidentified data analysed in this study are not publicly available due to institutional data‐sharing policies governing the JDDM registry. Data may be made available from the corresponding author upon reasonable request, subject to review and approval by the JDDM Study Group.
