# Supplementary material for: Within‐Person Seasonal Variability of Aminotransferases and Long‐Term Glycemic Control in Adults With Type 2 Diabetes (JDDM 85)
Source: Liver Int. 2026 Jun 19;46(7):e70743. doi: 10.1111/liv.70743 (PMC13282447; doi:10.1111/liv.70743)
Supplement: Supplementary file 1 — Figure S1: Estimated marginal means of monthly AST and ALT levels from random‐intercept linear mixed models. Figure S2: Cosinor fitted curves for AST and ALT (12‐month Cycle). Table S1: Baseline characteristics of the included and excluded patients. Table S2: Pre‐ and post‐imputation baseline patient characteristics. Table S3: Calendar distribution of monthly aminotransferase observations and final HbA1c measurements. Table S4: Seasonal variation of AST and ALT levels: Cosinor Regression Results. Table S5: Comparison of baseline patient characteristics across quartiles of seasonal AST amplitude. Table S6: Comparison of baseline patient characteristics across quartiles of seasonal ALT amplitude. Table S7: Sensitivity analysis: seasonal variation in abnormal ALT rates using sex‐specific thresholds. Table S8: Sensitivity analysis: within‐person ALT classification discordance using sex‐specific thresholds. Table S9: Association of seasonal amplitude quartiles of AST and ALT with final HbA1c. Table S10: Sensitivity analysis incorporating seasonal BMI amplitude. Table S11: Association between seasonal amplitude of liver enzymes and final HbA1c stratified by body mass index. Table S12: Stratified analysis by SGLT2 inhibitor use in the final year. Table S13: Sensitivity analyses for the association between seasonal aminotransferase amplitude and final HbA1c. [file LIV-46-0-s001.docx]

**Figure Legends**

**Supplementary Figure S1. Estimated marginal means of monthly AST and ALT levels from random-intercept linear mixed models.**

Least-squares means (with 95% confidence intervals) of (A) AST and (B) ALT for each calendar month, derived from random-intercept linear mixed models with month as a fixed (categorical) effect and patient identifier as a random intercept. Estimates were back-transformed from the log scale and are presented in IU/L. Error bars represent 95% confidence intervals. January serves as the reference month. Both enzymes showed a highly significant effect of month (P < 0.001), with peaks in late autumn to early winter and troughs in summer.

**Abbreviations:** ALT, alanine aminotransferase; AST, aspartate aminotransferase; CI, confidence interval; LMM, linear mixed model.

**
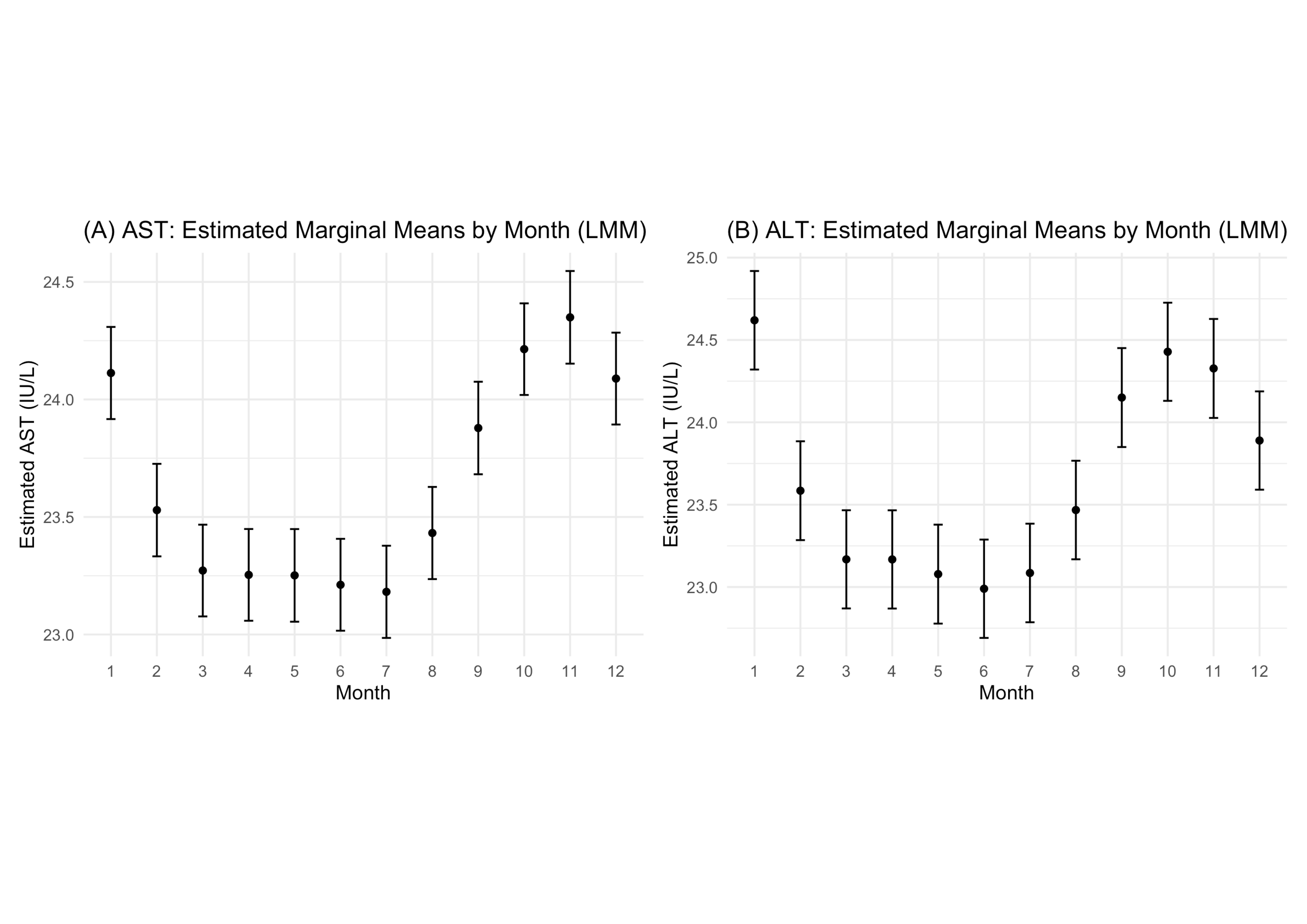
**

**Supplementary Figure S2. Cosinor Fitted Curves for AST and ALT (12-month Cycle).**

Fitted cosinor curves for (A) AST and (B) ALT showing the sinusoidal seasonal pattern over the 12-month cycle. Solid lines represent the fitted cosinor function, and dots represent observed monthly mean values. Both enzymes demonstrated significant sinusoidal seasonal patterns (P < 0.001), with estimated peaks in late autumn to early winter and troughs in mid-summer.

Abbreviations: ALT, alanine aminotransferase; AST, aspartate aminotransferase.**
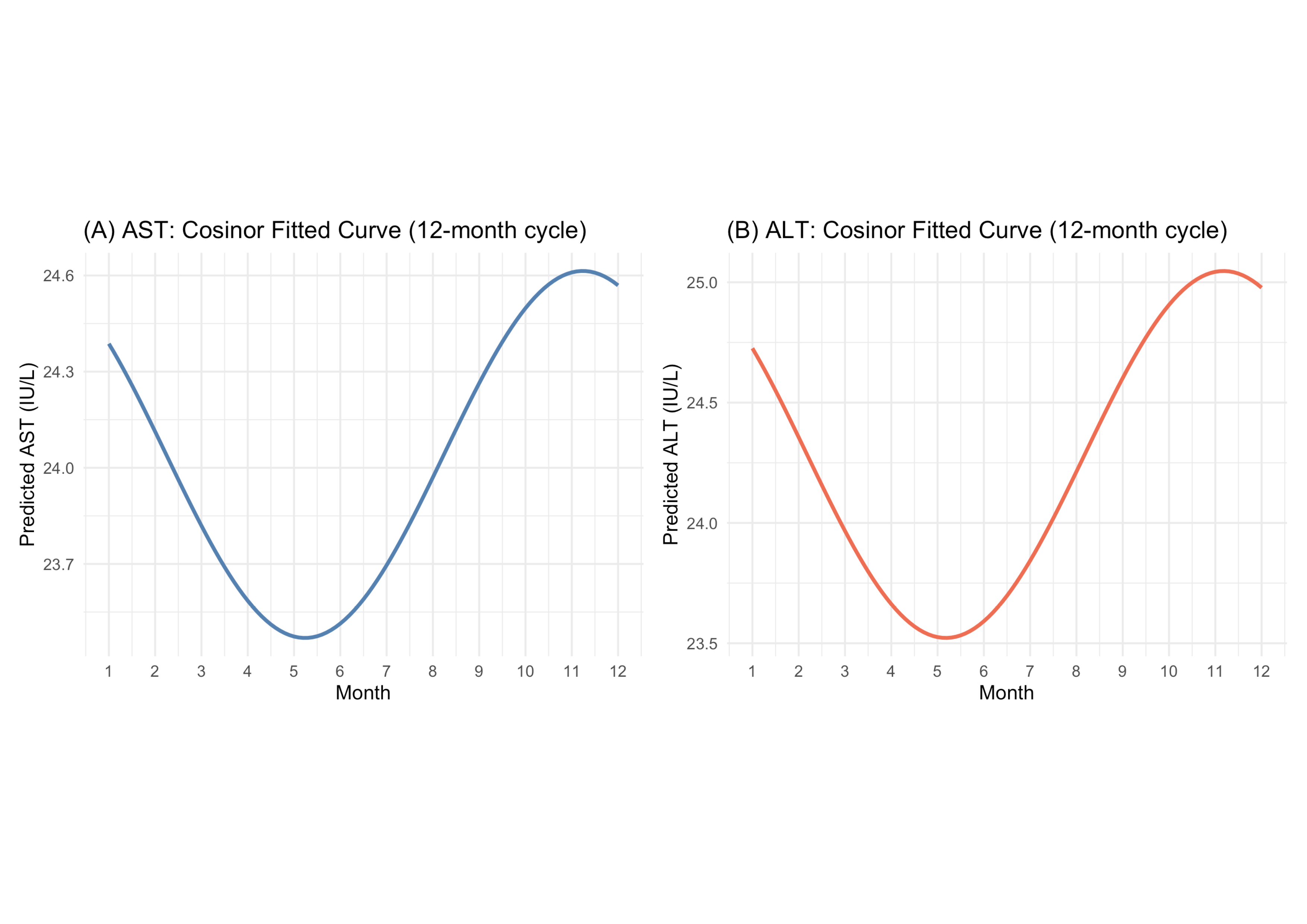
**

| **Supplementary Table S1. Baseline Characteristics of the Included and Excluded Patients** | | |
| --- | --- | --- |
| **Variable** | **Included**  n = 6039^1^ | **Excluded**  n = 3910^1^ |
| Age (Years) | 62.9 (9.9) | 63.6 (11.4) |
| Sex |  |  |
| Male | 3668 (61%) | 2525 (65%) |
| Female | 2371 (39%) | 1385 (35%) |
| Diabetes duration (Years) | 12.64 (8.58) | 12.6 (8.9) |
| Missing | 64 | 100 |
| Current alcohol drinking (Yes) | 1534 (26%) | 576 (16%) |
| Missing | 76 | 280 |
| Current smoking (Yes) | 855 (14%) | 341 (9.4%) |
| Missing | 76 | 280 |
| Baseline AST (IU/L) | 22 [19, 28] | 22 [18, 28] |
| Missing | 3106 | 2329 |
| Baseline ALT (IU/L) | 22 [16, 31] | 21 [15, 31] |
| Missing | 3054 | 1932 |
| Baseline BMI (kg/m²) | 25.5 (4.2) | 25.5 (4.5) |
| Missing | 1742 | 926 |
| Systolic BP (mmHg) | 128.9 (15.2) | 130.0 (16.1) |
| Missing | 1734 | 918 |
| Diastolic BP (mmHg) | 75.7 (12.8) | 75.8 (12.9) |
| Missing | 1734 | 918 |
| Baseline HbA1c, % (mmol/mol) | 7.1 (0.9) [54 (10)] | 7.2 (1.1) [55 (12)] |
| Missing | 1715 | 889 |
| Casual glucose level (mg/dL) | 156.1 (56.5) | 155.4 (60.8) |
| Missing | 2359 | 1091 |
| ¹ “Excluded” denotes patients removed due to ≥12 consecutive months of missing data; “Included” denotes the final analytic cohort.  Data are presented as the mean (SD), n (%), or median [Q1, Q3].  Abbreviations: BMI, body mass index; AST, aspartate aminotransferase; ALT, alanine aminotransferase; BP, blood pressure | | |

| **Supplementary Table S2. Pre- and Post-imputation Baseline Patient Characteristics** | | |
| --- | --- | --- |
| **Variable** | **Pre-imputation**  n = 6039^1^ | **Post-imputation**  n = 6039^1^ |
| Age (Years) | 62.9 (9.9) | 62.9 (9.9) |
| Sex |  |  |
| Male | 3668 (61%) | 3668 (61%) |
| Female | 2371 (39%) | 2371 (39%) |
| Current alcohol drinking (Yes) | 1534 (26%) | 1547 (26%) |
| Missing | 76 | 0 |
| Current smoking (Yes) | 855 (14%) | 863 (14%) |
| Missing | 76 | 0 |
| Baseline AST (IU/L) | 22 [19, 28] | 22 [18, 27] |
| Missing | 3106 | 0 |
| Baseline ALT (IU/L) | 22 [16, 31] | 21 [16, 30] |
| Missing | 3054 | 0 |
| Baseline BMI (kg/m²) | 25.5 (4.2) | 25.5 (4.2) |
| Missing | 1742 | 0 |
| ¹“Pre-imputation” columns reflect original incomplete data; “post-imputation” columns reflect one completed dataset (e.g., from multiple-imputation).  Data are presented as the mean (SD), n (%), or median [Q1, Q3].  Abbreviations: BMI, body mass index; AST, aspartate aminotransferase; ALT, alanine aminotransferase | | |

| **Supplementary Table S3. Calendar Distribution of Monthly Aminotransferase Observations and Final HbA1c Measurements** | | | |
| --- | --- | --- | --- |
| **Period** | **AST observations, n (%)** | **ALT observations, n (%)** | **Final HbA1c, n (%)** |
| **By month** |  |  |  |
| January | 18,387 (43.5) | 18,618 (44.0) | 2 (0.0) |
| February | 17,728 (41.9) | 17,957 (42.5) | 17 (0.3) |
| March | 19,132 (45.3) | 19,347 (45.8) | 22 (0.4) |
| April | 19,080 (45.1) | 19,294 (45.6) | 23 (0.4) |
| May | 17,533 (41.5) | 17,731 (41.9) | 20 (0.3) |
| June | 18,834 (44.6) | 18,992 (44.9) | 30 (0.5) |
| July | 18,349 (43.4) | 18,530 (43.8) | 30 (0.5) |
| August | 18,386 (43.5) | 18,560 (43.9) | 49 (0.8) |
| September | 17,623 (41.7) | 17,790 (42.1) | 81 (1.3) |
| October | 19,309 (45.7) | 19,496 (46.1) | 411 (6.8) |
| November | 17,491 (41.4) | 17,674 (41.8) | 1,604 (26.6) |
| December | 18,892 (44.7) | 19,057 (45.1) | 3,750 (62.1) |
| **By season** |  |  |  |
| Spring (Mar–May) | 55,745 (44.0) | 56,372 (44.5) | 65 (1.1) |
| Summer (Jun–Aug) | 55,569 (43.8) | 56,082 (44.2) | 109 (1.8) |
| Autumn (Sep–Nov) | 54,423 (42.9) | 54,960 (43.3) | 2,096 (34.7) |
| Winter (Dec–Feb) | 55,007 (43.4) | 55,632 (43.9) | 3,769 (62.4) |
| Percentages for AST and ALT represent the proportion of available patient-month observations out of the total possible (6,039 patients × number of months in the period).  Percentages for final HbA1c represent the proportion of patients (n = 6,039) whose final available HbA1c measurement was recorded in the indicated period.  Monthly observation rates ranged from 41.4% to 46.1% for AST and from 41.8% to 46.1% for ALT, with seasonal rates within 42.9%–44.5% across all four seasons, indicating no marked seasonal imbalance in measurement density.  **Abbreviations:** AST, aspartate aminotransferase; ALT, alanine aminotransferase; HbA1c, glycated hemoglobin. | | | |
|  | | | |
|  | | | |

| **Supplementary Table S4. Seasonal Variation of AST and ALT Levels: Cosinor Regression Results** | | | | |
| --- | --- | --- | --- | --- |
| **Outcome** | **Seasonal Component** | **Beta** | **95% CI** | **P value** |
| AST | Cosine | 0.0147 | 0.0128, 0.0166 | < 0.001 |
| AST | Sine | -0.0163 | -0.0182, -0.0144 | < 0.001 |
| ALT | Cosine | 0.0187 | 0.0158, 0.0216 | < 0.001 |
| ALT | Sine | -0.0207 | -0.0236, -0.0178 | < 0.001 |
| Cosinor regression (12-month periodicity) was applied to log-transformed AST and ALT values. Beta coefficients (95% CI) represent differences on the log scale relative to the annual mean. Abbreviations: AST, aspartate aminotransferase; ALT, alanine aminotransferase; CI, confidence interval | | | | |

| **Supplementary Table S5. Comparison of Baseline Patient Characteristics Across Quartiles of Seasonal AST Amplitude** | | | | | |
| --- | --- | --- | --- | --- | --- |
| **Characteristic** | **Q1 n = 1510** | **Q2 n = 1510** | **Q3 n = 1509** | **Q4 n = 1510** | **P value** |
| Sex |  |  |  |  | < 0.001 |
| Male | 854 (57%) | 908 (60%) | 953 (63%) | 953 (63%) |  |
| Female | 656 (43%) | 602 (40%) | 556 (37%) | 557 (37%) |  |
| Age (Years) | 63.5 (9.6) | 63.2 (9.8) | 62.8 (9.8) | 62.3 (10.2) | 0.011 |
| Diabetes duration (Years) | 13.3 (8.8) | 12.4 (8.5) | 12.6 (8.5) | 12.3 (8.5) | 0.004 |
| Missing | 21 | 13 | 13 | 17 |  |
| Current alcohol drinking (Yes) | 404 (27%) | 366 (24%) | 390 (26%) | 387 (26%) | 0.462 |
| Current smoking (Yes) | 233 (15%) | 201 (13%) | 224 (15%) | 205 (14%) | 0.285 |
| Baseline AST (IU/L) | 20 [18, 24] | 21 [18, 25] | 22 [19, 27] | 25 [20, 33] | < 0.001 |
| Baseline ALT (IU/L) | 19 [15, 26] | 20 [15, 27] | 22 [16, 31] | 26 [18, 40] | < 0.001 |
| Baseline BMI (kg/m²) | 25.1 (4.0) | 25.3 (3.9) | 25.6 (4.2) | 26.1 (4.6) | < 0.001 |
| Baseline systolic BP (mmHg) | 128.7 (14.7) | 128.8 (15.3) | 129.3 (15.3) | 128.7 (15.5) | 0.543 |
| Missing | 410 | 452 | 431 | 441 |  |
| Baseline diastolic BP (mmHg) | 74.4 (11.7) | 75.6 (13.2) | 76.3 (12.1) | 76.6 (14.1) | < 0.001 |
| Missing | 410 | 452 | 431 | 441 |  |
| Baseline HbA1c, % (mmol/mol) | 7.1 (0.9) [54 (10)] | 7.1 (0.9) [54 (10)] | 7.2 (1.0) [55 (11)] | 7.2 (1.1) [55 (12)] | 0.011 |
| Missing | 417 | 435 | 430 | 433 |  |
| Baseline casual glucose level (mg/dL) | 154.4 (54.6) | 154.0 (54.5) | 157.0 (55.7) | 158.9 (60.9) | 0.272 |
| Missing | 618 | 592 | 582 | 567 |  |
| Antihypertensive therapy (Yes) | 781 (52%) | 734 (49%) | 745 (49%) | 771 (51%) | 0.285 |
| Insulin therapy (Yes) | 290 (19%) | 307 (20%) | 314 (21%) | 329 (22%) | 0.362 |
| GLP-1 RA use (Yes) | 32 (2.1%) | 27 (1.8%) | 36 (2.4%) | 34 (2.3%) | 0.700 |
| SGLT2i use (Yes) | 4 (0.3%) | 0 (0%) | 1 (< 0.1%) | 3 (0.2%) | 0.172 |
| Data are presented as the mean (SD) or median [Q1, Q3] for continuous variables and as n (%) for categorical variables. “Missing” indicates the number of patients lacking data for that variable. P values test for differences across quartiles and are calculated using the Kruskal–Wallis test for continuous variables and Pearson’s Chi-squared or Fisher’s exact test for categorical variables.  Abbreviations: AST, aspartate aminotransferase; ALT, alanine aminotransferase; BMI, body mass index; BP, blood pressure; GLP-1 RA, glucagon-like peptide-1 receptor agonist; HbA1c, hemoglobin A1c; SGLT2i, sodium-glucose cotransporter-2 inhibitor | | | | | |

| **Supplementary Table S6. Comparison of Baseline Patient Characteristics Across Quartiles of Seasonal ALT Amplitude** | | | | | |  |
| --- | --- | --- | --- | --- | --- | --- |
| **Characteristic** | **Q1 n = 1510** | **Q2 n = 1510** | **Q3 n = 1509** | **Q4 n = 1510** | **P value** | |
| Sex |  |  |  |  | 0.941 | |
| Male | 923 (61%) | 923 (61%) | 909 (60%) | 913 (60%) |  | |
| Female | 587 (39%) | 587 (39%) | 600 (40%) | 597 (40%) |  | |
| Age (Years) | 63.2 (9.4) | 63.1 (10.0) | 62.4 (10.3) | 63.0 (9.9) | 0.390 | |
| Diabetes duration (Years) | 12.8 (8.6) | 12.8 (8.7) | 12.4 (8.4) | 12.5 (8.7) | 0.417 | |
| Missing | 16 | 15 | 20 | 13 |  | |
| Current alcohol drinking (Yes) | 394 (26%) | 414 (27%) | 368 (24%) | 371 (25%) | 0.186 | |
| Current smoking (Yes) | 226 (15%) | 227 (15%) | 218 (14%) | 192 (13%) | 0.228 | |
| Baseline AST (IU/L) | 21 [18, 25] | 22 [18, 26] | 22 [19, 27] | 23 [19, 30] | < 0.001 | |
| Baseline ALT (IU/L) | 20 [15, 27] | 20 [15, 29] | 22 [16, 31] | 24 [17, 35] | < 0.001 | |
| Baseline BMI (kg/m²) | 25.4 (4.1) | 25.4 (4.1) | 25.5 (4.2) | 25.7 (4.5) | 0.253 | |
| Baseline systolic BP (mmHg) | 128.9 (15.7) | 129.2 (14.5) | 128.9 (15.1) | 128.5 (15.6) | 0.967 | |
| Missing | 417 | 433 | 454 | 430 |  | |
| Baseline diastolic BP (mmHg) | 75.4 (12.5) | 75.5 (12.1) | 75.4 (12.1) | 76.6 (14.4) | 0.273 | |
| Missing | 417 | 433 | 454 | 430 |  | |
| Baseline HbA1c, % (mmol/mol) | 7.1 (0.9) [54 (10)] | 7.1 (0.9) [54 (10)] | 7.2 (1.0) [55 (11)] | 7.2 (1.0) [55 (11)] | 0.245 | |
| Missing | 413 | 430 | 448 | 424 |  | |
| Baseline casual glucose level (mg/dL) | 154.8 (55.3) | 155.3 (54.1) | 156.9 (57.7) | 157.4 (58.9) | 0.865 | |
| Missing | 606 | 601 | 594 | 558 |  | |
| Antihypertensive therapy (Yes) | 771 (51%) | 764 (51%) | 732 (49%) | 764 (51%) | 0.501 | |
| Insulin therapy (Yes) | 304 (20%) | 301 (20%) | 301 (20%) | 334 (22%) | 0.372 | |
| GLP-1 RA use (Yes) | 32 (2.1%) | 27 (1.8%) | 33 (2.2%) | 37 (2.5%) | 0.657 | |
| SGLT2i use (Yes) | 4 (0.3%) | 1 (< 0.1%) | 2 (0.1%) | 1 (< 0.1%) | 0.474 | |
| Data are presented as the mean (SD) or median [Q1, Q3] for continuous variables and as n (%) for categorical variables. “Missing” indicates the number of patients lacking data for that variable. P values test for differences across quartiles and are calculated using the Kruskal–Wallis test for continuous variables and Pearson’s Chi-squared or Fisher’s exact test for categorical variables. | | | | | |  |
| Abbreviations: AST, aspartate aminotransferase; ALT, alanine aminotransferase; BMI, body mass index; BP, blood pressure; GLP-1 RA, glucagon-like peptide-1 receptor agonist; HbA1c, hemoglobin A1c; SGLT2i, sodium-glucose cotransporter-2 inhibitor | | | | | |  |

| Supplementary Table S7. Sensitivity Analysis: Seasonal Variation in Abnormal ALT Rates Using Sex-Specific Thresholds | | | | | | | | |
| --- | --- | --- | --- | --- | --- | --- | --- | --- |
| **Season** | **ALT > 30 IU/L (primary, sex-independent)** | | | | **ALT sex-specific (M: 30 / F: 19 IU/L)** | | | |
|  | **Abnormal, %** | **OR** | **95% CI** | **P value** | **Abnormal, %** | **OR** | **95% CI** | **P value** |
| **Summer** | 20.6 | Ref. | Ref. | Ref. | 30.8 | Ref. | Ref. | Ref. |
| **Spring** | 20.8 | 1.02 | 0.99, 1.05 | 0.201 | 30.8 | 1.00 | 0.98, 1.03 | 0.697 |
| **Autumn** | 23.2 | 1.34 | 1.27, 1.41 | < 0.001 | 33.8 | 1.33 | 1.28, 1.39 | < 0.001 |
| **Winter** | 22.6 | 1.26 | 1.20, 1.32 | < 0.001 | 33.3 | 1.27 | 1.22, 1.32 | < 0.001 |
| Winter: December–February; Spring: March–May; Summer: June–August; Autumn: September–November.  Summer is set as the reference season. Odds ratios (OR) and 95% confidence intervals (CI) were estimated from mixed-effects logistic regression with patient as a random intercept. Estimates were pooled across five multiply imputed datasets using Rubin’s rules.  The primary analysis used a sex-independent threshold of 30 IU/L based on the AASLD 2023 practice guidance. The sex-specific analysis applied thresholds of 30 IU/L for men and 19 IU/L for women, as proposed by Prati et al.  **Abbreviations:** ALT, alanine aminotransferase; CI, confidence interval; OR, odds ratio. | | | | | | | | |

| Supplementary Table S8. Sensitivity Analysis: Within-Person ALT Classification Discordance Using Sex-Specific Thresholds | | |
| --- | --- | --- |
| **Classification** | **ALT > 30 IU/L (primary), n (%)** | **ALT sex-specific (M:30/F:19 IU/L), n (%)** |
| **All patients** | n = 6,039 | n = 6,039 |
| Concordant normal | 4,678 (77.5) | 3,814 (63.2) |
| Concordant abnormal | 1,057 (17.5) | 1,791 (29.7) |
| **Discordant classification** | 303 (5.0) | 434 (7.2) |
| **Borderline subgroup** | n = 2,672 | n = 3,718 |
| **Discordant classification** | 303 (11.4) | 434 (11.7) |
| For each patient, the mean of all available winter (December–February) and summer (June–August) ALT measurements across the entire observation period was computed, and each seasonal mean was classified as abnormal or normal using the applicable threshold. Only patients with measurements in both seasons were included. Counts and percentages were averaged across five multiply imputed datasets.  Concordant normal: both seasonal means at or below the threshold. Concordant abnormal: both seasonal means above the threshold. Discordant: winter-mean and summer-mean classifications differed, regardless of direction.  The borderline subgroup was defined as patients whose annual mean ALT fell within ±10 IU/L of the applicable threshold (20–40 IU/L for the 30 IU/L threshold; 9–29 IU/L for the 19 IU/L female threshold; 20–40 IU/L for the 30 IU/L male threshold).  **Abbreviations:** ALT, alanine aminotransferase. | | |

| **Supplementary Table S9. Association of Seasonal Amplitude Quartiles of AST and ALT with Final HbA1c** | | | | | |
| --- | --- | --- | --- | --- | --- |
|  | **Quartile** | **N** | **Amplitude, median [IQR], IU/L** | **β (95% CI)** | **P value** |
| AST | Q1 | 1,510 | 2.9 [2.4-3.5] | Reference | - |
|  | Q2 | 1,510 | 4.0 [3.3-4.8) | 0.05 (-0.01, 0.11) | 0.081 |
|  | Q3 | 1,509 | 5.1 [4.3-6.3] | 0.09 (0.03, 0.15) | 0.004 |
|  | Q4 | 1,510 | 7.6 [5.8-10.8] | 0.15 (0.08, 0.21) | <0.001 |
|  | ***P for trend*** |  |  |  | **<0.001** |
| ALT | Q1 | 1,510 | 3.7 [2.8-5.1] | Reference | - |
|  | Q2 | 1,510 | 5.0 [3.8-7.1] | -0.01 (-0.07, 0.05) | 0.692 |
|  | Q3 | 1,509 | 6.6 [4.9-9.4] | -0.00 (-0.06, 0.06) | 0.909 |
|  | Q4 | 1,510 | 9.5 [6.6-14.5] | 0.05 (-0.01, 0.11) | 0.080 |
|  | ***P for trend*** |  |  |  | **0.081** |
| Seasonal amplitude was calculated as peak-to-trough difference from STL decomposition of log-transformed monthly values. Quartiles were defined based on log-transformed amplitude; median and IQR are presented in IU/L for clinical interpretation. Q1 represents patients with lowest seasonal variation (reference). β coefficients indicate the difference in final HbA1c (%) compared with Q1. Models were adjusted for baseline transferases levels (log-transformed), sex, age, baseline HbA1c, baseline BMI, alcohol use, smoking status, diabetes duration, and follow-up duration. **Abbreviations:** AST, aspartate aminotransferase; ALT, alanine aminotransferase; CI, confidence interval; IQR, interquartile range | | | | | |

| **Supplementary Table S10.** **Sensitivity Analysis Incorporating Seasonal BMI Amplitude** | | | | | | |
| --- | --- | --- | --- | --- | --- | --- |
|  | **AST Sensitivity (BMI amplitude added)** | | | **ALT Sensitivity (BMI amplitude added)** | | |
| **Characteristic** | **Beta** | **95% CI**^1^ | **P value** | **Beta** | **95% CI**^1^ | **P value** |
| **AST/ALT amplitude (SD)** | **0.06** | **0.04, 0.08** | **<0.001** | **0.02** | **0.00, 0.04** | **0.040** |
| BMI amplitude (SD) | 0.00 | -0.02, 0.03 | 0.666 | 0.01 | -0.01, 0.03 | 0.445 |
| Baseline AST/ALT (2014-01) | -0.09 | -0.18, 0.00 | 0.043 | 0.01 | -0.05, 0.07 | 0.818 |
| Sex (Male) | 0.07 | 0.02, 0.11 | 0.004 | 0.07 | 0.02, 0.11 | 0.005 |
| Age | -0.01 | -0.01, 0.00 | <0.001 | -0.01 | -0.01, 0.00 | <0.001 |
| First measured HbA1c | 0.34 | 0.32, 0.36 | <0.001 | 0.34 | 0.32, 0.36 | <0.001 |
| Baseline BMI | 0.01 | 0.01, 0.02 | <0.001 | 0.01 | 0.01, 0.02 | <0.001 |
| Current alcohol drinking (Yes) | 0.01 | -0.03, 0.06 | 0.469 | 0.01 | -0.03, 0.05 | 0.485 |
| Current smoking (Yes) | -0.02 | -0.05, 0.02 | 0.328 | -0.02 | -0.05, 0.02 | 0.319 |
| Diabetes duration (Years) | 0.01 | 0.01, 0.01 | <0.001 | 0.01 | 0.01, 0.01 | <0.001 |
| Follow-up months | 0.00 | -0.01, 0.00 | 0.342 | 0.00 | -0.01, 0.00 | 0.358 |
| ^1^Coefficients (95% CI) represent changes in final HbA1c (%) per 1-SD increment in enzyme amplitude or BMI amplitude, adjusting for baseline enzyme levels, demographic variables, and follow-up duration.  This table shows regression results for AST and ALT amplitude after adding a seasonal BMI amplitude term to the model.  Abbreviations: AST, aspartate aminotransferase; ALT, alanine aminotransferase; BMI, body mass index; CI, confidence interval | | | | | | |

| **Supplementary Table S11.** **Association between seasonal amplitude of liver enzymes and final HbA1c stratified by body mass index** | | | | | | | |
| --- | --- | --- | --- | --- | --- | --- | --- |
|  | | **BMI < 25 kg/m² (N=3012)** | | | **BMI ≥ 25 kg/m² (N=3027)** | | |
| **Group** | **Characteristic** | **Beta** | **95% CI^1^** | **P value** | **Beta** | **95% CI^1^** | **P value** |
| AST Stratified Models | **AST amplitude (SD)** | **0.03** | **0.00, 0.06** | **0.038** | **0.10** | **0.06, 0.13** | **<0.001** |
|  | Baseline AST (2014-01) | -0.03 | -0.14, 0.07 | 0.546 | -0.15 | -0.27, -0.03 | 0.012 |
|  | Sex (Male) | 0.08 | 0.03, 0.14 | 0.003 | 0.06 | -0.02, 0.13 | 0.130 |
|  | Age | 0.00 | 0.00, 0.00 | 0.377 | -0.01 | -0.02, -0.01 | <0.001 |
|  | First measured HbA1c | 0.32 | 0.29, 0.35 | <0.001 | 0.34 | 0.31, 0.38 | <0.001 |
|  | Baseline BMI | 0.01 | 0.00, 0.03 | 0.048 | 0.01 | 0.00, 0.02 | 0.291 |
|  | Current alcohol drinking (Yes) | -0.01 | -0.06, 0.04 | 0.569 | 0.04 | -0.03, 0.11 | 0.233 |
|  | Current smoking (Yes) | -0.01 | -0.05, 0.03 | 0.641 | -0.02 | -0.08, 0.03 | 0.389 |
|  | Diabetes duration (years) | 0.01 | 0.00, 0.01 | <0.001 | 0.01 | 0.01, 0.01 | <0.001 |
|  | Follow-up months | 0.00 | -0.02, 0.01 | 0.627 | 0.00 | -0.02, 0.01 | 0.517 |
| ALT Stratified Models | **ALT amplitude (SD)** | **-0.01** | **-0.03, 0.02** | **0.626** | **0.05** | **0.02, 0.09** | **0.002** |
|  | Baseline ALT (2014-01) | 0.04 | -0.03, 0.11 | 0.298 | -0.03 | -0.11, 0.06 | 0.532 |
|  | Sex (Male) | 0.08 | 0.03, 0.14 | 0.003 | 0.05 | -0.02, 0.13 | 0.154 |
|  | Age | 0.00 | 0.00, 0.00 | 0.363 | -0.01 | -0.02, -0.01 | <0.001 |
|  | First measured HbA1c | 0.32 | 0.29, 0.35 | <0.001 | 0.35 | 0.31, 0.38 | <0.001 |
|  | Baseline BMI | 0.01 | 0.00, 0.03 | 0.065 | 0.01 | 0.00, 0.02 | 0.296 |
|  | Current alcohol drinking (Yes) | -0.02 | -0.07, 0.03 | 0.516 | 0.04 | -0.03, 0.11 | 0.237 |
|  | Current smoking (Yes) | -0.01 | -0.05, 0.03 | 0.649 | -0.02 | -0.08, 0.03 | 0.374 |
|  | Diabetes duration (Years) | 0.01 | 0.00, 0.01 | <0.001 | 0.01 | 0.01, 0.01 | <0.001 |
|  | Follow-up months | 0.00 | -0.02, 0.01 | 0.651 | 0.00 | -0.02, 0.01 | 0.529 |
| ¹Beta coefficients (95% CI) reflect changes in final HbA1c (%) per 1-SD increment in enzyme amplitude, adjusting for baseline enzyme and key covariates.  Participants were stratified by baseline BMI using the Japanese obesity criterion (low BMI: <25 kg/m²; high BMI: ≥25 kg/m²). Models were adjusted for baseline enzyme levels, sex, age, baseline HbA1c, alcohol consumption, smoking status, diabetes duration, and follow-up months.  Abbreviations: ALT, alanine aminotransferase; AST, aspartate aminotransferase; BMI, body mass index; CI, confidence interval; HbA1c, glycated hemoglobin; SD, standard deviation. | | | | | | | |

| **Supplementary Table S12. Stratified Analysis by SGLT2 Inhibitor Use in the Final Year** | | | | | | | |
| --- | --- | --- | --- | --- | --- | --- | --- |
|  | | **SGLT2i On in 2020** | | | **SGLT2i Off in 2020** | | |
| **Group** | **Characteristic** | **Beta** | **95% CI**^1^ | **P value** | **Beta** | **95% CI**^1^ | **P value** |
| AST stratified models | **AST amplitude (SD)** | **0.04** | **0.00, 0.08** | **0.029** | **0.07** | **0.04, 0.10** | **<0.001** |
|  | Baseline AST (2014-01) | -0.09 | -0.21, 0.04 | 0.182 | -0.10 | -0.20, 0.01 | 0.063 |
|  | Sex (Male) | 0.05 | -0.03, 0.14 | 0.194 | 0.07 | 0.02, 0.13 | 0.007 |
|  | Age | -0.01 | -0.01, 0.00 | <0.001 | 0.00 | -0.01, 0.00 | 0.002 |
|  | First measured HbA1c | 0.26 | 0.23, 0.29 | <0.001 | 0.38 | 0.36, 0.41 | <0.001 |
|  | Baseline BMI | 0.01 | 0.00, 0.02 | 0.008 | 0.01 | 0.00, 0.01 | 0.105 |
|  | Current alcohol drinking (Yes) | 0.01 | -0.06, 0.08 | 0.809 | 0.02 | -0.03, 0.07 | 0.489 |
|  | Current smoking (Yes) | -0.02 | -0.07, 0.04 | 0.504 | -0.02 | -0.06, 0.02 | 0.346 |
|  | Diabetes duration (years) | 0.01 | 0.01, 0.02 | <0.001 | 0.01 | 0.00, 0.01 | <0.001 |
|  | Follow-up months | 0.00 | -0.02, 0.02 | 0.928 | -0.01 | -0.02, 0.00 | 0.178 |
| ALT stratified models | **ALT amplitude (SD)** | **0.02** | **-0.02, 0.06** | **0.245** | **0.02** | **0.00, 0.05** | **0.063** |
|  | Baseline ALT (2014-01) | 0.00 | -0.09, 0.08 | 0.913 | 0.00 | -0.08, 0.08 | 0.949 |
|  | Sex (Male) | 0.05 | -0.03, 0.14 | 0.208 | 0.07 | 0.02, 0.12 | 0.010 |
|  | Age | -0.01 | -0.01, 0.00 | <0.001 | 0.00 | -0.01, 0.00 | <0.001 |
|  | First measured HbA1c | 0.26 | 0.23, 0.29 | <0.001 | 0.39 | 0.36, 0.41 | <0.001 |
|  | Baseline BMI | 0.01 | 0.00, 0.02 | 0.009 | 0.01 | 0.00, 0.01 | 0.103 |
|  | Current alcohol drinking (Yes) | 0.01 | -0.07, 0.08 | 0.849 | 0.02 | -0.03, 0.07 | 0.491 |
|  | Current smoking (Yes) | -0.02 | -0.07, 0.04 | 0.503 | -0.02 | -0.06, 0.02 | 0.333 |
|  | Diabetes duration (Years) | 0.01 | 0.01, 0.02 | <0.001 | 0.01 | 0.00, 0.01 | <0.001 |
|  | Follow-up months | 0.00 | -0.02, 0.02 | 0.916 | -0.01 | -0.02, 0.00 | 0.191 |
| ¹Beta coefficients (95% CI) reflect changes in final HbA1c (%) per 1-SD increment in enzyme amplitude, adjusting for baseline enzyme and key covariates.  The analyses are stratified by SGLT2 inhibitor (SGLT2i) status at 2020 (“On” vs. “Off”) to examine whether AST or ALT amplitude associations with final HbA1c differ by late-stage SGLT2i use.  Abbreviations: AST, aspartate aminotransferase; ALT, alanine aminotransferase; CI, confidence interval; BMI, body mass index | | | | | | | |

| **Supplementary Table S13. Sensitivity Analyses for the Association Between Seasonal Aminotransferase Amplitude and Final HbA1c** | | | | | | | | |
| --- | --- | --- | --- | --- | --- | --- | --- | --- |
| **Sensitivity Analysis** |  | **AST amplitude** | | |  | **ALT amplitude** | | |
|  | **N** | **β** | **95% CI** | **P value** | **N** | **β** | **95% CI** | **P value** |
| **Primary multivariable analysis (Model 2)** | 6,039 | 0.062 | 0.039, 0.084 | < 0.001 | 6,039 | 0.023 | 0.002, 0.045 | 0.032 |
| Only participants with ≥42 monthly aminotransferase observations | 2,158 | 0.080 | 0.041, 0.119 | < 0.001 | 2,218 | 0.036 | -0.001, 0.072 | 0.055 |
| Observed-data cosinor amplitude (no imputation) | 5,793 | 0.047 | 0.025, 0.069 | < 0.001 | 5,794 | 0.026 | 0.004, 0.048 | 0.019 |
| Adjusted for mean enzyme level over follow-up | 6,039 | 0.050 | 0.027, 0.073 | < 0.001 | 6,039 | 0.010 | -0.011, 0.032 | 0.344 |
| Adjusted for season of final HbA1c measurement | 6,039 | 0.061 | 0.039, 0.084 | < 0.001 | 6,039 | 0.023 | 0.002, 0.044 | 0.033 |
| Split-period analysis (amplitude 2014–2017, outcome 2018–2020) | 6,034 | 0.043 | 0.019, 0.067 | < 0.001 | 6,034 | 0.004 | -0.018, 0.026 | 0.722 |
| β coefficients (95% CI) represent the change in final HbA1c (%) per 1-SD increment in seasonal amplitude.  Primary multivariable analysis (Model 2): the reference model included standardized seasonal amplitude (1 SD), baseline AST/ALT (log-transformed, January 2014), sex, age, first measured HbA1c, baseline BMI, drinking status, smoking status, diabetes duration, and follow-up duration as covariates.  Only participants with ≥42 monthly aminotransferase observations: cohort restricted to patients with at least 42 observed monthly aminotransferase measurements out of 84 follow-up months. Multiple imputation was applied to the remaining missing values.  Observed-data cosinor amplitude (no imputation): individual seasonal amplitude was estimated by fitting a 12-month cosinor regression to observed log-transformed monthly values for each patient with at least 12 observed measurements, without imputation.  Adjusted for mean enzyme level over follow-up: baseline AST/ALT (January 2014) was replaced by the log-transformed mean AST/ALT over the entire follow-up period.  Adjusted for season of final HbA1c measurement: the season of final HbA1c measurement was added as an additional covariate to the main model.  Split-period analysis: seasonal amplitude was calculated from monthly values during 2014–2017, and final HbA1c was assessed during 2018–2020.  N values are shown as AST / ALT separately when sample sizes differed between enzymes due to differential observation completeness.  **Abbreviations:** AST, aspartate aminotransferase; ALT, alanine aminotransferase; CI, confidence interval; HbA1c, glycated hemoglobin; SD, standard deviation. | | | | | | | | |
